# Encephalopathy in COVID-19 Presenting With Acute Aphasia Mimicking Stroke

**DOI:** 10.3389/fneur.2020.587226

**Published:** 2020-10-19

**Authors:** Umberto Pensato, Lorenzo Muccioli, Elena Pasini, Maria Tappatà, Lorenzo Ferri, Lilia Volpi, Laura Licchetta, Stella Battaglia, Giada Rossini, Isabella Bon, Maria Carla Re, Luigi Cirillo, Luigi Simonetti, Laura Ludovica Gramegna, Roberto Michelucci, Pietro Cortelli, Andrea Zini, Francesca Bisulli

**Affiliations:** ^1^Department of Biomedical and NeuroMotor Sciences, University of Bologna, Bologna, Italy; ^2^IRCCS Istituto delle Scienze Neurologiche di Bologna, Bologna, Italy^‡^; ^3^Microbiology Unit, DIMES, University of Bologna, Bologna, Italy

**Keywords:** cytokine release syndrome, car-t, ICANS, neurology, delirium, SARS-CoV-2, encephalitis

## Abstract

**Introduction:** Neurological manifestations are emerging as relatively frequent complications of corona virus disease 2019 (COVID-19), including stroke and encephalopathy. Clinical characteristics of the latter are heterogeneous and not yet fully elucidated, while the pathogenesis appears related to neuroinflammation in a subset of patients.

**Case:** A middle-aged man presented with acute language disturbance at the emergency department. Examination revealed expressive aphasia, mild ideomotor slowing, and severe hypocapnic hypoxemia. Multimodal CT assessment and electroencephalogram (EEG) did not reveal any abnormalities. COVID-19 was diagnosed based on chest CT findings and positive severe acute respiratory syndrome coronavirus 2 (SARS-CoV-2) reverse transcription PCR (RT-PCR) on nasopharyngeal swab. The following day, neurological symptoms progressed to agitated delirium and respiratory status worsened, requiring admission to the ICU and mechanical ventilation. Brain MRI and cerebrospinal fluid (CSF) studies were unremarkable. RT-PCR for SARS-CoV-2 on CSF was negative. He received supportive treatment and intravenous low-dose steroids. His neurological and respiratory status resolved completely within 2 weeks.

**Conclusions:** We report a patient with reversible COVID-19-related encephalopathy presenting as acute aphasia, mimicking stroke or status epilepticus, eventually evolving into delirium. Although large-vessel stroke is frequently encountered in COVID-19, our case suggests that focal neurological deficits may occur as the earliest feature of encephalopathy. Neurological status reversibility and the absence of abnormalities on brain MRI are consistent with a functional rather than a structural neuronal network impairment.

## Introduction

Coronavirus disease 2019 (COVID-19) is characterized predominantly by respiratory symptoms, although neurological manifestations are increasingly described, including encephalopathy and stroke (1, 2). A higher incidence of large artery ischemic stroke has been reported, also in young patients without cardiovascular risk factors (3). Coagulopathy and endothelial dysfunction secondary to an infection-induced systemic inflammatory response likely play a major role (4). The pathogenesis of encephalopathy remains more debated. A role of neuroinvasion by severe acute respiratory syndrome coronavirus 2 (SARS-CoV-2) is not supported in most cases (1, 5, 6), whereas indirect effects related to hypoxemia, multiple organ dysfunction syndrome, and cytokine storm are suggested as potentially underlying causes (7). Hereby, we report the case of a middle-aged man who was thoroughly investigated for acute expressive aphasia mimicking stroke or status epilepticus, eventually evolving to encephalopathy with a self-limited course.

## Case

A hypertensive, dyslipidemic, right-handed 54-years-old man with a 1-week history of sore throat, dysgeusia, and hyposmia presented with acute language disturbance developed within 1 h at the emergency department.

**Figure 1:**
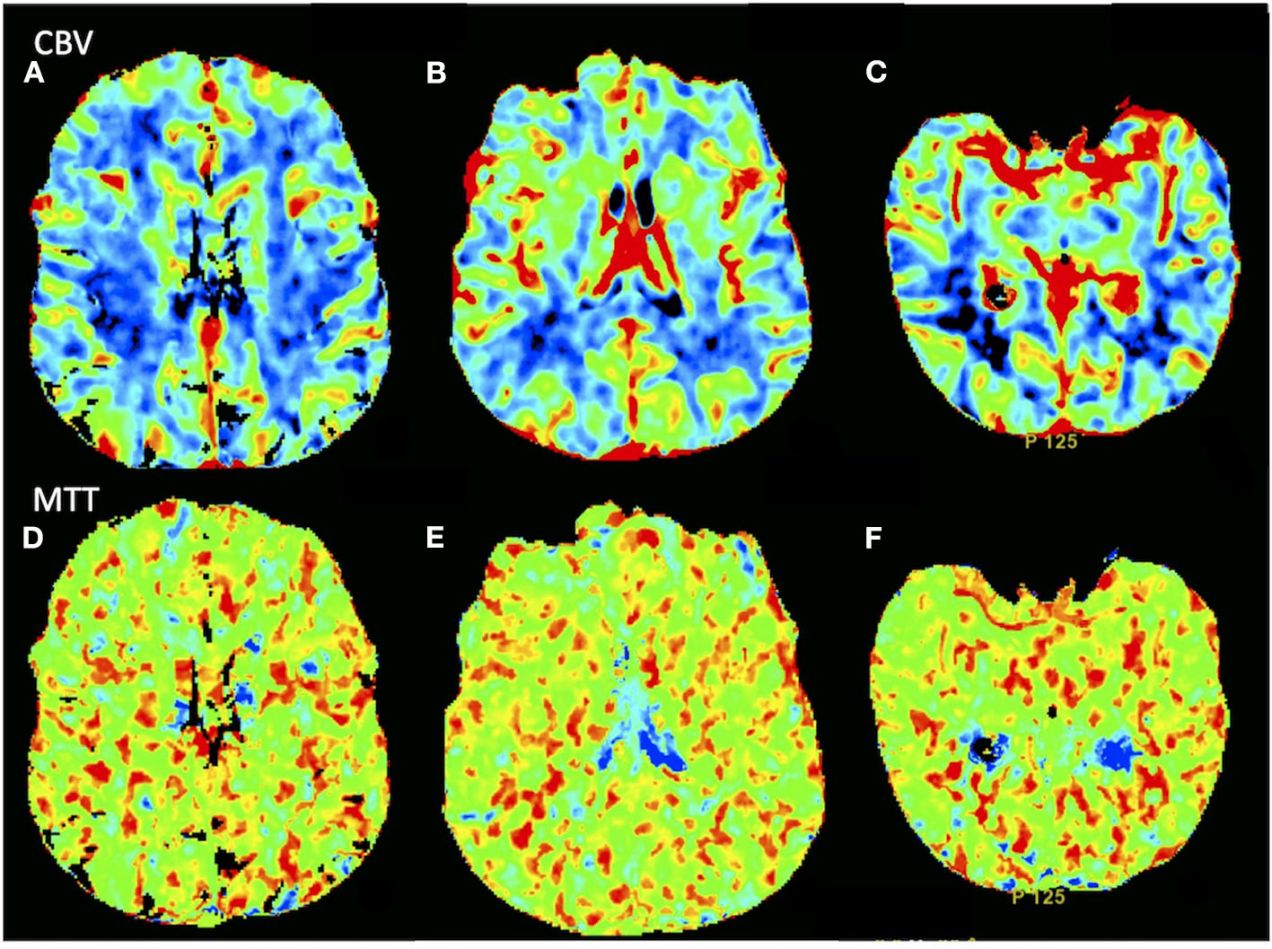
Computed tomography perfusion showed no asymmetry in **(A–C)** CBV and **(D–F)** MTT parametric maps. CBV, cerebral blood volume; MTT, mean transit time.

**Figure 2:**
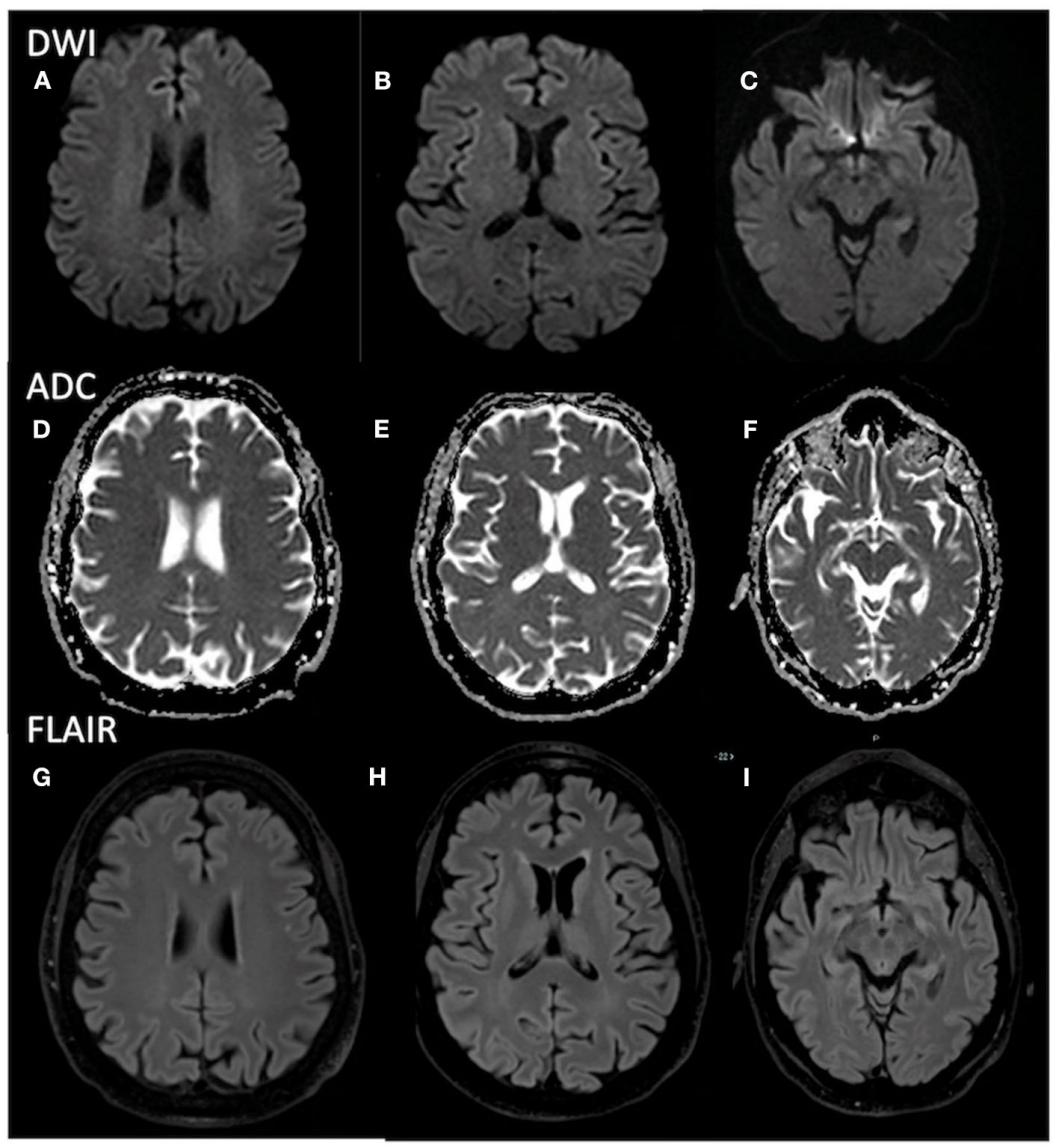
Brain MRI shows no abnormalities in **(A–C)** DWI, **(D–F)** ADC maps, and **(G–I)** FLAIR images. MRI, magnetic resonance imaging; DWI, diffusion weighted imaging; ADC, apparent diffusion coefficient; FLAIR, fluid-attenuated inversion recovery.

Body temperature was 37.5°C. Neurological examination revealed expressive aphasia and mild ideomotor slowing without further cognitive alterations. His speech was slow, effortful, and contained phonological and neological paraphasias, whereas comprehension was preserved. Suspecting a stroke, the patient underwent multimodal CT assessment, which was unrevealing (Figure 1). Electroencephalogram (EEG) and brain MRI were also unremarkable. Arterial blood gas analysis and blood tests showed hypocapnic hypoxemia (PaO_2_/FiO_2_ = 252), elevated D-dimer level (3.32 mg/L), lymphopenia, and elevated inflammatory markers (Supplementary Table 1). COVID-19 was diagnosed on the basis of bilateral interstitial pneumonia on chest CT and positive RT-PCR for SARS-CoV-2 on nasopharyngeal swab. Cerebrospinal fluid (CSF) examination showed proteins 41 mg/dl, glucose 62 mg/dl, and nucleated cells 2/mmc. Microbiology analysis of CSF was unremarkable, including negative RT-PCR for SARS-CoV-2, herpesvirus DNA [herpes simplex virus types 1 and 2 (HSV1, HSV2), cytomegalovirus (CMV), Epstein–Barr virus (EBV), human herpesvirus 6 (HHV6), and varicella zoster virus (VZV)], enterovirus DNA, Gram stain, and bacterial culture. The patient was hospitalized and started on oxygen therapy, hydroxychloroquine, low-molecular-weight heparin, intravenous low-dose steroids, and antibiotics. The following day, neurological symptoms progressed to agitated delirium, and his respiratory status worsened, requiring admission to the ICU. The patient was sedated and placed on mechanical ventilation for 5 days. Subsequently, he appeared oppositional and disinhibited, while his language disturbance resolved, with the exception of rare anomia. In the following days, both his respiratory and neurological status progressively improved, normalizing in 7 days. A follow-up EEG and brain MRI performed 10 days after aphasia onset (Figure 2) did not show any abnormalities. After 60 days, the patient referred only mild dysgeusia and was otherwise asymptomatic.

## Discussion

Our patient presented with acute expressive aphasia mimicking an acute stroke, lasting >24 h, in the context of symptoms related to SARS-CoV-2 infection. He had cardiovascular risk factors and elevated D-dimer levels at presentation. COVID-19 has been associated with an increased incidence of stroke (2, 8, 9), which may also represent the presenting manifestation (3, 10). Large-vessel ischemic strokes in COVID-19 patients have been reported to occur in multiple vascular territories, in young patients without cardiovascular risk factors, and in patients with prophylactic anticoagulation. Likely, the virus-related systemic inflammation leads to vascular endothelial dysfunction and coagulopathy. Accordingly, COVID-19 patients who develop ischemic stroke usually showed markedly elevated D-dimer levels and more severe systemic involvement compared with COVID-19 patients without cerebrovascular events (11). Surprisingly, multimodal CT assessment studies did not reveal any focal hypoperfusion area or artery vessel abnormalities in our patient, and two brain MRI definitively excluded stroke or structural lesions. A focal status epilepticus (“aphasic status”) was also excluded, based on the absence of EEG abnormalities (12).

Neurological status rapidly progressed to encephalopathy; thus, we interpreted his language disturbance as an early focal feature of a generalized central nervous system (CNS) involvement. Encephalopathy in COVID-19 has been shown to have various manifestations with ranging severities, characterized by subacute onset and fluctuations or progressive course, eventually reversible (5). Although language disturbances have already been described in this syndrome as early features (13), an acute presentation mimicking stroke has never been reported so far. Encephalopathy in the contest of potentially neurotropic virus infection, such as SARS-CoV-2, may raise suspicion of a viral encephalitis; however, CSF and MRI findings in our patient were inconsistent with this hypothesis, in line with the vast majority of COVID-19 patients presenting with central neurological manifestations (1, 5, 6). Accordingly, clinical responses observed to various immunomodulatory treatments, such as corticosteroids (13, 14) and plasmapheresis (15), suggest an immune-mediated pathogenesis, at least for a subgroup of patients. Recently, cytokine-mediated neuroinflammation has been proposed as the underlying pathogenesis of COVID-19-related encephalopathy/encephalitis (7, 13, 16), a peculiar pathogenic mechanism also responsible for immune effector cell-associated neurotoxicity syndrome (ICANS) (17–20). This is a neuropsychiatric complication of chimeric antigen receptor T-cell therapy, which also shares clinical features with encephalopathy related to COVID-19 (20) and is typically associated with cytokine release syndrome (18). Interestingly, expressive aphasia often represents the first manifestation of neurotoxicity (18, 19), and a case series reported language impairment as the earliest feature in 19/22 patients who later developed severe ICANS (17). In this disease, aphasia usually develops over hours/days, although, as in our patient, language symptoms may present also acutely mimicking stroke (19). In our patient, focal neurological symptoms presented without a concomitant brain lesion, suggesting a functional rather than structural neuronal network impairment, as in ICANS. Some limitations to this report should be noted. Indeed, we did not test repetition, reading and writing at first evaluation, and did not perform frontal assessment batteries; thus, we could not rule out with certainty that the language disturbance was secondary to bradypsychia related to an incoming global encephalopathy. However, at presentation, there were no signs of frontal lobe syndrome or encephalopathy such as fluctuating/decreased attention or level of consciousness. Thus, his neurological presentation was clinically indistinguishable from acute onset expressive aphasia caused by cerebrovascular diseases, requiring a comprehensive diagnostic evaluation for stroke.

## Conclusions

We report a patient with COVID-19-related encephalopathy presenting with acute aphasia, mimicking stroke. Although large-vessel stroke is frequently encountered in COVID-19, our case suggests that COVID-19-related encephalopathy may present with acute language disturbances as the earliest feature. Neurologists should be aware of this clinical presentation, as it may have an impact on the differential diagnosis and management of these patients.

## Ethics Statement

Ethical review and approval was not required for the study on human participants in accordance with the local legislation and institutional requirements. The patients/participants provided their written informed consent to participate in this study. Written informed consent was obtained from the patient for the publication of any potentially identifiable images or data included in this article.

## Author Contributions

UP and LM drafted the manuscript. All authors listed have made a substantial, direct and intellectual contribution to the work, and approved it for publication.

## Conflict of Interest

The authors declare that the research was conducted in the absence of any commercial or financial relationships that could be construed as a potential conflict of interest.

## Abbreviations

CRS: cytokine releasing syndrome
CAR: chimeric antigen receptor
ICANS: immune-effector-associated neurotoxicity syndrome.
