# Supplementary material for: Encephalopathy in COVID-19 Presenting With Acute Aphasia Mimicking Stroke
Source: Front Neurol. 2020 Oct 19;11:587226. doi: 10.3389/fneur.2020.587226 (PMC7604480; doi:10.3389/fneur.2020.587226)
Supplement: Supplementary file 1 [file Table_1.DOCX]

Supplementary Material

**Supplementary Table 1 – Blood tests**

|  | *Reference values* | *1^st^ day* | 6^th^ day | 16^th^ day (discharge day) |
| --- | --- | --- | --- | --- |
| **Hematology**  White-cell count (10^9^/L)  Neutrophil count (10^9^/L)  Lymphocyte count (10^9^/L)  Hemoglobin (g/dL)  Platelet count (10^9^/L) | 3.6-10.5  1.5-7.7  1.1-4  12.0-15.6  160-370 | 6.25  4.75  0.96  13.6  280 | 17.94  16.42  0.96  11.0  420 | 16.55  13.09  2.23  10.4  318 |
| **Coagulation**  INR  aPTT  Fibrinogen (mg/dL)  D-dimer mg/L | <1.2  0.82-1.25  150-400  <0.55 | 1.09  0.83  494  3.32 | 1.08  0.92  490  1.45 | 1.18  0.99  368  2.21 |
| **Biochemistry**  Sodium (mmol/L)  Potassium (mmol/L)  Glucose (mg/dL)  Urea (mg/dL)  Creatinine (mg/dL)  eGFR (mL/min)  Total protein (g/dL)  Albumin (g/L)  Total bilirubin (mg/dL)  AST (U/L)  ALT (U/L) | 136-145  3.5-5.3  60-110  17-43  0.5-1.2  NA  6.6-8.3  35-50  <1.2  <35  <35 | 136  3.7  97  26  0.87  98  5.3  28.6  0.75  72  76 | 147  4.1  156  60  0.72  106  5.2  25.7  0.35  37  51 | 140  3.7  67  NA  .73  105  5.8  NA  1.22  62  149 |
| **Serum inflammatory proteins**  C-reactive protein (mg/dL)  IL-6 (pg/mL)  Procalcitonin (ng/mL)  Ferritin (ng/mL)  LDH (U/L) | <0.5  <5.9  <0.5  11-306  <248 | 4.75  17.3  <0.1  574  326 | 7.59  13.6  <0.1  509  275 | 1.86  120 (o 21.1)  <0.1  461  457 |
